# Identification of Lck-derived peptides applicable to anti-cancer vaccine for patients with human leukocyte antigen-A3 supertype alleles

**DOI:** 10.1038/sj.bjc.6604071

**Published:** 2007-11-27

**Authors:** M Naito, Y Komohara, Y Ishihara, M Noguchi, Y Yamashita, T Shirakusa, A Yamada, K Itoh, M Harada

**Affiliations:** 1 Department of Immunology, Kurume University School of Medicine, 67 Asahi-machi, Kurume, Fukuoka 830-0011, Japan; 2 Center of the 21st Century COE Program for Medical Science, Kurume University School of Medicine, 67 Asahi-machi, Kurume, Fukuoka 830-0011, Japan; 3 Cancer Vaccine Department Division, Kurume University Research Center for Innovative Cancer Therapy, Kurume University School of Medicine, 67 Asahi-machi, Kurume, Fukuoka 830-0011, Japan; 4 Department of Urology, Kurume University School of Medicine, 67 Asahi-machi, Kurume, Fukuoka 830-0011, Japan; 5 Department of Surgery, Fukuoka University School of Medicine, 45-1, 7-chome Nanakuma, Fukuoka, Fukuoka 814-0180, Japan

**Keywords:** Lck, cytotoxic T lymphocyte, peptide, HLA-A3 supertype

## Abstract

The identification of peptide vaccine candidates to date has been focused on human leukocyte antigen (HLA)-A2 and -A24 alleles. In this study, we attempted to identify cytotoxic T lymphocyte (CTL)-directed Lck-derived peptides applicable to HLA-A11^+^, -A31^+^, or -A33^+^ cancer patients, because these HLA-A alleles share binding motifs, designated HLA-A3 supertype alleles, and because the Lck is preferentially expressed in metastatic cancer. Twenty-one Lck-derived peptides were prepared based on the binding motif to the HLA-A3 supertype alleles. They were first screened for their recognisability by immunoglobulin G (IgG) in the plasma of prostate cancer patients, and the selected candidates were subsequently tested for their potential to induce peptide-specific CTLs from peripheral blood mononuclear cells of HLA-A3 supertype^+^ cancer patients. As a result, four Lck peptides were frequently recognised by IgGs, and three of them – Lck_90−99_, Lck_449−458_, and Lck_450−458_ – efficiently induced peptide-specific and cancer-reactive CTLs. Their cytotoxicity towards cancer cells was mainly ascribed to HLA class I-restricted and peptide-specific CD8^+^ T cells. These results indicate that these three Lck peptides are applicable to HLA-A3 supertype^+^ cancer patients, especially those with metastasis. This information could facilitate the development of peptide-based anti-cancer vaccine for patients with alleles other than *HLA-A2* and *-A24*.

Many tumour antigens and their peptides that are recognised by cytotoxic T lymphocytes (CTLs) have been identified from a variety of cancer types (Renkvist *et al*, 2001), and subsequent clinical applications, including a form of peptide-based immunotherapy, have been performed (Yamada and Itoh, 2006). Most of these peptides are limited to human leukocyte antigen (HLA)-A2 or -A24 alleles, primarily because of the higher worldwide frequency of these alleles (Imanishi *et al*, 1992). Several HLA alleles are known to have structural similarities according to peptide-binding motif analyses, and four supertypes have been proposed: HLA-A2, -A3, -B7, and -B44 supertype alleles (Sette and Sidney, 1999). Among them, the A3 supertype alleles include the allelic products of at least five common HLA-A alleles, including A3, A11, A31, A33, and A68. Thirty-eight percent of Caucasians, 53% of Chinese, 46% of Japanese, and 43% of North American African Americans and Hispanics were positive for the HLA-A3 supertype alleles (Sette and Sidney, 1999). Therefore, the identification of peptide vaccine candidates for HLA-A3 supertype^+^ cancer patients could facilitate the development of peptide-based immunotherapy for many ethnic populations.

The Lck protein (p56^*lck*^), the *src* family tyrosine kinase, is known to be essential for both T-cell development and function (Veillette *et al*, 1989). It is notable that the Lck protein is aberrantly expressed in several malignancies, including colon carcinoma, small cell lung carcinoma, and prostate carcinoma with a trend of preferential expression in metastatic lesions (Robinson *et al*, 1996; McCracken *et al*, 1997; Krystal *et al*, 1998; Lutz *et al*, 1998). Although biological roles of the Lck protein in cancer cells have not been fully confirmed, several lines of evidence suggest that this protein contributes to the process of neoplastic transformation (Marth *et al*, 1988; Robinson *et al*, 1996; McCracken *et al*, 1997; Krystal *et al*, 1998; Lutz *et al*, 1998). In support of this idea, several studies have reported that the Lck protein may contribute to the anchorage-independent growth of TGF-*β*-initiated tumour cells through the transcription of p56^*lck*^ with a type I promoter (Amundadottir and Leder, 1998). In addition, we previously reported that the Lck-derived peptides can be recognised by cancer-reactive CTLs of cancer patients, and that Lck peptide-specific CTLs can be induced from patients with distant metastases, but not from those without distant metastases (Harashima *et al*, 2001). In this study, we further attempted to identify novel Lck-derived peptide candidates that would be applicable to cancer patients with HLA-A3 supertype alleles to expand the possibilities of a peptide-based vaccine for metastatic cancer patients with alleles other than HLA-A2 and -A24.

## MATERIALS AND METHODS

### Patients

Peripheral blood mononuclear cells (PBMCs) were obtained from prostate cancer patients who had provided written informed consent. These patients included HLA-A11^+^, -A31^+^, and -A33^+^ patients, but PBMCs from HLA-A3^+^ or -A68.1^+^ patients were not included because of their extremely low frequency (1.6 and 0.5%) in the Japanese population (Aizawa, 1986). None of the subjects were infected with HIV. Twenty millilitres of peripheral blood was obtained, and PBMCs were prepared by Ficoll–Conray density gradient centrifugation. All of the samples were cryopreserved until they were used for the experiments. The expression of HLA-A11, -A31, and -A33 molecules on PBMCs was determined by flow cytometry using the following antibodies: anti-HLA-A11 monoclonal antibody (mAb) (cat. no. 0284HA; One Lambda Inc., Canoga, CA, USA), anti-HLA-A31 mAb (cat. no. 0273HA; One Lambda), and anti-HLA-A33 mAb (cat. no. 0612HA; One Lambda).

### Peptides

The Lck-derived peptides that are provided in Table 1 were prepared on the basis of the binding motifs to the HLA-A11, -A31, and -A33 molecules (Parker *et al*, 1994). All peptides were of >90% purity and were purchased from the Biologica Co. (Nagoya, Japan). Epstein–Barr virus (EBV)-derived, tyrosinase-related protein 2 (TRP-2)-derived, and HIV-derived peptides were used as controls binding to HLA-A3 supertype alleles. All peptides were dissolved with dimethyl sulphoxide at a dose of 10 mg ml^−1^.

### Measurement of IgGs reactive to Lck peptides

The levels of immunoglobulin Gs (IgGs) reactive to Lck peptides were measured by the Luminex™ method, as reported previously (Komatsu *et al*, 2004). In brief, 100 *μ*l of diluted plasma was incubated with 5 *μ*l of colour-coded beads (Luminex Corp., Austin, TX, USA) coated with each of the Lck peptides on 96-well filter plates (MABVN1250; Millipore Corp., Bedford, MA, USA) for 2 h at room temperature on a plate shaker. The plates were then washed with Tween-PBS and incubated with 100 *μ*l of biotin-conjugated goat anti-human IgG (BA-3080: Vector Laboratories, Burlingame, CA, USA) for 1 h at room temperature on a plate shaker. After the plates were washed, 100 *μ*l of streptavidin-PE was added to the wells, and the samples were incubated for 30 min at room temperature on a plate shaker. The bound beads were washed four times, and 100 *μ*l of Tween-PBS was added to each well. Fifty microlitres of sample was examined using the Luminex system.

### Induction of peptide-specific CTLs from PBMCs

The assay for the detection of peptide-specific CTLs was performed according to a previously reported method with several modifications (Hida *et al*, 2002). In brief, PBMCs (1 × 10^5^ cells per well) were incubated with 10 *μ*l ml^−1^ of each peptide in quadruplicate in a U-bottom-type 96-well microculture plate (Nunc, Roskilde, Denmark) in 200 *μ*l of culture medium. The culture medium consisted of 45% RPMI 1640, 45% AIM-V medium (Gibco BRL, Gaithersburg, MD, USA), 10% FCS, 100 U ml^−1^ of interleukin-2 (IL-2), and 0.1 mM MEM nonessential amino-acid solution (Gibco BRL). Every 3 or 4 days, half the culture medium was removed and replaced by new medium containing the corresponding peptide (20 *μ*g ml^−1^) and 100 U ml^−1^ IL-2. On the fourteenth day of the culture, the cultured cells were separated into four wells. Two wells were used for the culture with the corresponding peptide-pulsed C1R-A11, -A31, or -A33 cells, and the other two were used for the culture with HIV peptide-pulsed C1R-A11, -A31, or -A33 cells. After an 18-h incubation, the supernatant was collected, and the level of interferon (IFN)-*γ* was determined by enzyme-linked immunosorbent assay. The successful induction of peptide-specific CTLs was judged to be positive when a value of *P*<0.05 was reached by a two-tailed Student's *t*-test.

### Cell lines

SQ-1 is an HLA-A11^+^ lung carcinoma cell line. LC-1 is an HLA-A31^+^ and HLA-A33^+^ lung carcinoma cell line. COLO 201 and LNCaP are HLA-A3 supertype negative colon carcinoma and prostate carcinoma cell lines, respectively. All tumour cell lines were maintained in RPMI 1640 (Invitrogen) with 10% FCS. The expression of the Lck protein on these cell lines was examined by flow cytometry using anti-Lck mAb (mouse IgG2b) (sc-433; Santa Cruz Biotechnology Inc., Santa Cruz, CA, USA), followed by FITC-conjugated goat anti-mouse IgG no. 55493, Cappel ICN, Aurora, OH, USA). Normal mouse IgG (sc-3879; Santa Cruz Biotechnology Inc.) was used as a control for anti-Lck mAb.

### Cytotoxicity assay

Peptide-stimulated PBMCs were tested for their cytotoxicity against COLO 201 (HLA-A2^+^), SQ-1 (HLA-A11^+^), and LC-1 (HLA-A31^+^/A33^+^) by a standard 6-h ^51^Cr-release assay. Phytohaemagglutinin (PHA)-activated T cells were used as a negative control. Two thousand ^51^Cr-labelled cells per well were cultured with effector cells in 96-round well plates at the indicated effector/target ratio. The specific ^51^Cr release was calculated according to the following formula: (test c.p.m.−spontaneous c.p.m.). Spontaneous release was determined by the supernatant of the sample incubated with no effector cells, and the total release was then determined by the supernatant of the sample incubated with 1% Triton X (Wako Pure Chemical Industries, Osaka, Japan). In some experiments, CD8^+^ T cells were positively isolated using a CD8 Positive Isolation Kit (Dynal, Oslo, Norway). An measure of 10 *μ*g ml^−1^ of either anti-HLA class I (W6/32: mouse IgG2a) or anti-HLA-DR (L243: mouse IgG2a) mAb was added into wells at the initiation of the culture.

### Cold inhibition assay

The specificity of peptide-stimulated CTLs against cancer cells was confirmed by a cold inhibition assay. In brief, ^51^Cr-labelled target cells (2 × 10^3^ cells per well) were cultured with the effector cells (2 × 10^4^ cells per well) in 96-round well plates with 2 × 10^4^ cold target cells. C1R-A11, -A31, and -A33, which were pre-pulsed with either the HIV peptide or a corresponding peptide, were used as cold target cells.

### Statistics

The statistical significance of the data was determined using a two-tailed Student's *t*-test. A *P*-value of less than 0.05 was considered statistically significant.

## RESULTS

### Measurement of IgGs reactive to the Lck peptides in the plasma of cancer patients

First, we prepared 21 peptides derived from the Lck protein based on the binding motifs to the HLA-A3 supertype alleles (Table 1). These include 9- and 10-mer peptides. Although the A3 supertype alleles include HLA-A3 and -A68, we preferentially considered the binding capacity to HLA-A11, -A31, and -A33 molecules because the HLA-A3 and -A68 alleles are very rare in the Japanese population (Aizawa, 1986). We first screened these peptide candidates based on their recognisability by the IgGs of prostate cancer patients, since we previously observed that IgGs reactive to CTL-directed peptides are detectable in the plasma of patients with different types of cancer (Nakatsura *et al*, 2002; Ohkouchi *et al*, 2002). In addition, the number of available PBMCs from prostate cancer patients was limited, and 21 peptides was too large a number of candidates to individually test their potential to generate peptide-specific CTLs from the PBMCs of cancer patients. The results were that IgGs reactive to the Lck_90−99_, Lck_449−458_, Lck_450−458_, and Lck_452−461_ peptides were detected in the plasma of 9, 11, 7, and 5 out of the 20 prostate cancer patients, respectively (Table 2). Immunoglobulin Gs reactive to the other 17 Lck peptides were less frequently observed in the plasma of cancer patients (data not shown).

### Induction of peptide-specific CTLs from the PBMCs of prostate cancer patients with HLA-A3 supertype alleles

We next determined whether or not these four Lck peptides, which were more frequently recognised by IgGs in cancer patients than were the other 17 peptides, could induce peptide-specific CTLs from the PBMCs of HLA-A11^+^, -A31^+^, or -A33^+^ prostate cancer patients. The PBMCs were stimulated *in vitro* with each of the Lck-derived peptides or with a control peptide. The peptide-stimulated PBMCs were then assessed for their IFN-*γ* production in response to corresponding peptide-pulsed C1R-A11, -A31, or -A33 cells. The results of 17 patients (seven with HLA-A11, five with HLA-A31, and five with HLA-A33) are shown in Table 3. Successful induction of peptide-specific CTLs was judged to be positive when the *P-*value was <0.05. As a result, the Lck_90−99_, Lck_449−458_, Lck_450−458_, and Lck_452−461_ peptides induced corresponding peptide-specific CTLs from the PBMCs of five, two, four, and three out of seven HLA-A11^+^ cancer patients. These peptides induced peptide-specific CTLs from the PBMCs of three, three, four, and one out of five HLA-A31^+^ cancer patients, and of two, three, three, and one out of five HLA-A33^+^ cancer patients, respectively. In total, the Lck_90−99_, Lck_449−458_, Lck_450−458_, and Lck_452−461_ peptides induced corresponding peptide-specific CTLs from the PBMCs of 10, 8, 11, and 5 out of 17 HLA-A3 supertype^+^ cancer patients, respectively. These rates of peptide-specific CTL induction were comparable with those of the positive control EBV and TRP-2 peptides. These results suggest that the Lck_90−99_, Lck_449−458_, and Lck_450−458_ peptides have the potential to efficiently generate peptide-specific CTLs in the PBMCs of prostate cancer patients with HLA-A3 supertype alleles.

### Induction of cancer-reactive CTLs from the PBMCs of prostate cancer patients with HLA-A3 supertype alleles

**Figure 1:**
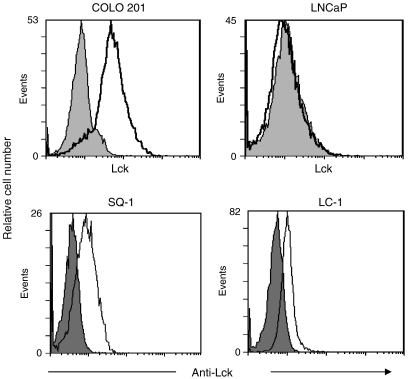
The expression of the Lck protein in four tumour cell lines. These tumour cell lines were analysed by flow cytometry for their expression of the Lck protein. These cells were stained first with anti-Lck mAb, followed by staining with FITC-conjugated anti-mouse IgG mAb. The grey background represents staining first with normal mouse IgG, followed by staining with FITC-conjugated anti-mouse IgG mAb.

Before the cytotoxicity assay, we selected target cancer cells that express the HLA-A3 supertype alleles and the Lck protein. Flow cytometric analysis revealed that HLA-A3 supertype negative colon carcinoma COLO 201, HLA-A11^+^ lung carcinoma SQ-1, and HLA-A31^+^/A33^+^ lung carcinoma LC-1 were positive for the Lck protein (Figure 1). Although we had established several LNCaP transfectants that express each of HLA-A11, -A31, and -A33 molecules (Minami *et al*, 2007), the LNCaP cell line was negative for the Lck protein. Therefore, we used SQ-1 as an Lck-expressing HLA-A11^+^ target, and LC-1 as an Lck-expressing HLA-A31^+/−^A33^+^ target. COLO 201 was used as an Lck-expressing HLA-A3 supertype negative target.

**Figure 2:**
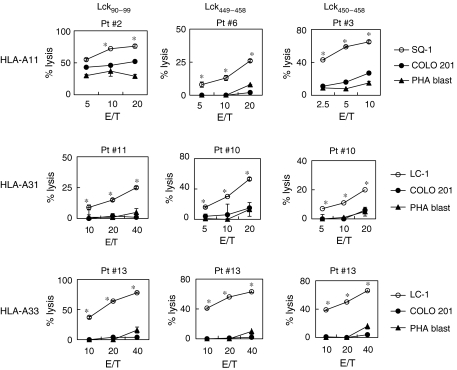
Cytotoxicity of peptide-stimulated PBMCs from HLA-A3 supertype^+^ prostate cancer patients. Peptide-stimulated PBMCs from HLA-A3 supertype^+^ prostate cancer patients were tested for their cytotoxicity towards three different targets by a 6-h ^51^Cr-release assay. Phytohaemagglutinin (PHA)-stimulated T-cell blasts from HLA-A3 supertype^+^ healthy donors were used as a control. ^*^Statistically significant at *P*<0.05.

We then determined whether or not the CTLs induced by *in vitro* stimulation with each of the Lck_90−99_, Lck_449−458_, and Lck_450−458_ peptides could show cytotoxicity against cancer cells (Figure 2). The PBMCs from HLA-A11^+^ patients (patients 2, 6, and 3), which were stimulated *in vitro* with each of the Lck_90−99_, Lck_449−458_, and Lck_450−458_ peptides, exhibited higher levels of cytotoxicity against HLA-A11^+^ SQ-1 cells than against HLA-A11^−^ COLO 201 cells and HLA-A11^+^ PHA-stimulated T-cell blasts. Similarly, these peptides possessed the ability to induce LC-1 (HLA-A31^+^/-A33^+^)-reactive CTLs from the PBMCs of HLA-A31^+^ and -A33^+^ patients (patients 10, 11, and 13). Each of the peptide-specific CTLs showed higher levels of cytotoxicity against LC-1 cells than against COLO 201 cells or T-cell blasts. Taken together, these results indicate that the PBMCs that were stimulated *in vitro* with each of the Lck_90−99_, Lck_449−458_, and Lck_450−458_ peptides can exhibit cytotoxicity against cancer cells in an HLA-A11-, -A31-, or -A33-restricted manner.

### Peptide-specific and CD8^+^ T-cell-dependent cytotoxicity against cancer cells

**Figure 3:**
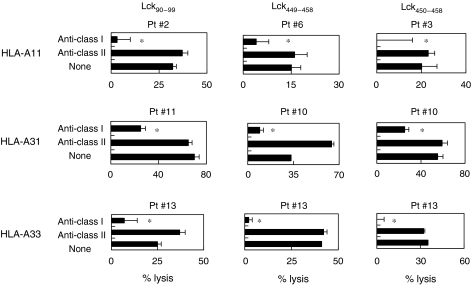
Human leukocyte antigen class I-restricted cytotoxicity of peptide-stimulated PBMCs against cancer cells. Purified CD8^+^ T cells from peptide-stimulated PBMCs of HLA-A3 supertype^+^ patients were tested for their cytotoxicity against HLA-A11^+^ SQ-1 cells or HLA-A31^+^/A33^+^ LC-1 cells in the presence of the indicated mAbs by a 6-h ^51^Cr-release assay. ^*^Statistically significant at *P*<0.05.

**Figure 4:**
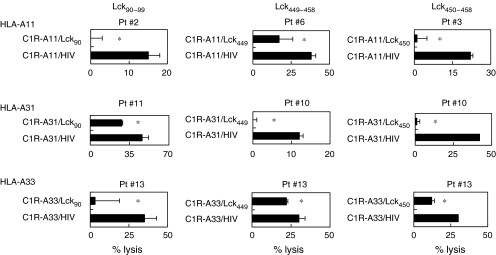
Peptide-specific cytotoxicity against cancer cells. Purified CD8^+^ T cells from peptide-stimulated PBMCs of HLA-A3 supertype^+^ patients were tested for their cytotoxicity against HLA-A11^+^ SQ-1 cells or HLA-A31^+^/A33^+^ LC-1 cells in the presence of unlabelled C1R-A11, -A31, or -A33 cells, which were pre-loaded with either the corresponding peptide or the HIV peptide, by a 6-h ^51^Cr-release assay. ^*^Statistically significant at *P*<0.05.

We further attempted to identify the cells responsible for the cytotoxicity of Lck peptide-stimulated PBMCs. Purified CD8^+^ T cells were used in the following experiments. As shown in Figure 3, the cytotoxicity of purified CD8^+^ T cells from the peptide-stimulated PBMCs against SQ-1 and LC-1 was significantly decreased by the addition of anti-HLA class I mAb, but not by the addition of anti-HLA class II (HLA-DR). In addition, the cytotoxicity of these cells against SQ-1 and LC-1 was significantly inhibited by the addition of corresponding peptide-pulsed unlabelled C1R-A11, C1R-A31, and C1R-A33 cells, but not by the addition of HIV peptide-pulsed unlabelled C1R-A11, C1R-A31, or C1R-A33 cells (Figure 4). These results suggested that the cytotoxicity of peptide-stimulated PBMCs against Lck-expressing cancer cells was mainly dependent on HLA class I-restricted and Lck peptide-specific CD8^+^ T cells.

## DISCUSSION

Although the Lck protein is known to be essential for both T-cell development and function (Veillette *et al*, 1989), this protein is aberrantly expressed in several malignancies, including colon carcinoma, small cell lung carcinoma, and prostate carcinoma with a trend of preferential expression in metastatic lesions (Robinson *et al*, 1996; McCracken *et al*, 1997; Krystal *et al*, 1998; Lutz *et al*, 1998). We previously reported that HLA-A24-restricted tumour-reactive CTLs from patients with distant metastases can recognise Lck-derived peptides as a tumour antigen (Harashima *et al*, 2001). We also identified Lck-derived peptides that were applicable to metastatic cancer patients positive for HLA-A2 molecules (Imai *et al*, 2001). Thereafter, we carried out peptide-based immunotherapy against various types of cancer in which Lck-derived peptides were vaccinated into HLA-A24^+^ or -A2^+^ cancer patients (Sato *et al*, 2003; Mine *et al*, 2004). The primary purpose of the present study was to identify new Lck-derived peptides that would be applicable to patients with HLA-A3 supertype alleles to expand the possibilities for peptide-based anti-cancer vaccine for many ethnic populations, as described in the Introduction.

We first screened 21 Lck peptide candidates based on their ability to be recognised by the IgGs of cancer patients before testing their ability to induce HLA class I-restricted CTLs, because this approach was successful in our previous studies (Matsueda *et al*, 2005; Minami *et al*, 2007). As a result, we selected four Lck peptides that were frequently recognised by IgG. These candidates were then tested for their ability to induce peptide-specific CTLs from PBMCs of HLA-A3 supertype^+^ cancer patients, and three of them were found to effectively induce peptide-specific and cancer-reactive CTLs. Although we did not include peptides that were recognised by IgG less frequently in the CTL induction assay in this study, we previously included them and compared to those that were recognised by IgG more frequently. As a result, we observed a correlation between their recognisability by IgG and the induction rate of CTLs (Harada *et al*, 2004; Matsueda *et al*, 2005). In our clinical trials, peptide vaccination frequently resulted in the induction of IgGs reactive to the administered CTL-directed peptides, and the induction of IgGs reactive to vaccinated peptides was positively correlated with clinical responses and with the survival of vaccinated patients (Sato *et al*, 2003; Mine *et al*, 2004). Peptides that can be recognised by both CTLs and IgG might be more useful in peptide-based immunotherapy than those that are recognised only by CTLs.

Although we selected three Lck peptides from 21 candidates, the most important point was to determine whether or not the selected candidates could have the potential to induce cancer-reactive CTLs in cancer patients. In this study, we demonstrated that three Lck peptides, Lck_90−99_, Lck_449−458_, and Lck_450−458_, could induce cancer-reactive CTLs from the PBMCs of HLA-A3 supertype^+^ cancer patients. In addition, a cold inhibition assay revealed that the cytotoxicity towards HLA-A3 supertype^+^ cancer cells was mainly dependent on Lck peptide-specific CD8^+^ T cells. These lines of evidence indicate that these Lck peptides would be useful as a peptide-based anti-cancer vaccine for HLA-A3 supertype^+^ cancer patients.

The optimal COOH-terminal amino acid of A11-binding peptides is lysine, and that of A31- or A33-binding peptides is arginine (Rammensee *et al*, 1995; Sidney *et al*, 1996). Among the three Lck peptides identified in this study to be candidates for HLA-A3 supertype alleles, the Lck_90−99_ peptide carries lysine at the COOH terminus, and both the Lck_449−458_ and Lck_450−458_ peptides carry arginine at the COOH terminus. However, our results revealed that the Lck_90−99_ peptide induced peptide-specific CTLs from the PBMCs of HLA-A31^+^ or -A33^+^ cancer patients and, on the other hand, both the Lck_449−458_ and Lck_450−458_ peptides induced peptide-specific CTLs from the PBMCs of HLA-A11^+^ cancer patients. We reported a similar observation in our previous study (Matsueda *et al*, 2005). These observations might suggest that peptides carrying lysine or arginine at the COOH terminus fit the binding motif for all HLA-A11, -A31, and -A33 molecules.

In conclusion, we identified three new peptide candidates that were applicable to HLA-A3 supertype^+^ cancer patients. In combination with known Lck peptides for HLA-A2^+^ or -A24^+^ cancer patients (Harashima *et al*, 2001; Imai *et al*, 2001), those identified in the present study enable us to develop a peptide-based anti-cancer vaccine for cancer patients with metastases in diverse ethnic populations.

## Figures and Tables

**Table 1:** Summary of Lck-derived peptide candidates binding to the HLA-A3 supertype alleles

			**Binding score^a^**				
**Peptides**	**Amino-acid sequence**	**Bind to^b^**	**A3**	**A11**	**A31**	**A33**	**A68**
27–35	IVRLDGKGR		0.2	0.2	1	15	200
36–45	LLIRNGSEVR		6	0.12	4	9	10
37–45	LIRNGSEVR		0.4	0.08	2	15	5
90–99	ILEQSGEWWK		60	0.8	1	0.3	4.5
131–139	NLSRKDAER		4	0.08	2	9	7.5
146–154	NTHGSFLIR		1.8	0.4	4	3	50
198–207	TFPGLHELVR		0.012	0.08	1.2	3	0.75
290–299	NLMKQLQHQR		6	0.16	6	9	10
291–299	LMKQLQHQR		4	0.08	2	15	5
293–302	KQLQHQRLVR		1.08	1.08	24	0.9	10
294–302	QLQHQRLVR		8	0.16	4	9	5
354–363	FIEERNYIHR		1.2	0.16	4	15	7.5
379–387	KIADFGLAR		7.2	0.48	12	4.5	10
388–397	LIEDNEYTAR		0.4	0.08	2	15	5
429–438	LLTEIVTHGR		9	0.08	2	9	5
430–438	LTEIVTHGR		0.3	0.2	1	3	50
449–458	VIQNLERGYR		0.12	0.08	2	15	5
450–458	IQNLERGYR		0.036	0.12	2	3	5
452–461	NLERGYRMVR		24	0.16	4	9	5
471–480	QLMRLCWKER		3	0.08	3	9	10
472–480	LMRLCWKER		2	0.04	1	15	
EBV	IVTDFSVIK	A11	10.0	4.0	0.6	0.5	240
Flu	NVKNLYEKVK	A11	3.0	1.0	0.1	0.5	180
TRP-2	LLGPGRPYR	A31/A33	6.0	0.1	2.0	9.0	15
HIV	RLRDLLLIVTR	A31	—	—	—	—	—

EBV=Epstein–Barr virus; HLA=human leukocyte antigen; TRP-2=tyrosinase-related protein 2.

a The peptide-binding score was calculated based on the predicted half time of dissociation from HLA class I molecules as obtained from a website (Bioinformatics and Molecular Analysis Section, Computational Bioscience and Engineering Laboratory, Division of Computer Research and Technology, NIH). The binding score of the HIV peptide was not calculated because the peptide consisted of 11-mer amino acids.

b Previously reported HLA class I alleles in which the peptides have immunogenicity are shown.

**Table 2:** IgG reactive to Lck peptides in the plasma of prostate cancer patients

**Patient**	**Lck_90–99_**	**Lck_449–458_**	**Lck_450–458_**	**Lck_452–461_**	**No peptide**
*Immunofluorescence intensity*					
A	49	48	36	32	47
B	**99**	**113**	**98**	87	64
C	**66**	**74**	**56**	43	26
D	33	**132**	63	40	46
E	**92**	**123**	**96**	**82**	47
F	15	16.5	12	10	7
G	**1044**	**1361**	**1138**	**1096**	447
H	54	69	48	55	62
I	**100**	**86**	**75**	**134**	49
J	44	52	37	34	36
K	**64**	**65**	46	40	24
L	36	43	33	29	20
M	**319**	**274**	**228**	**213**	80
N	**3415**	**3489**	**3206**	**3217**	1672
O	23	25	19	15	13
P	45	49	35	33	21
Q	52	**55**	44	40	37
R	42	53	37	41	49
S	**81**	**79**	59	53	53
T	146	178	115	121	253
					
Positive/total	9/20	11/20	7/20	5/20	

IgG=immunoglobulin G.

We measured the levels of peptide-specific IgG in the plasma of 20 patients. The fluorescence intensity of the plasma (1 : 100 dilution) was measured by the Luminex method. Positive results (>no peptide × 1.5) are shown in bold.

**Table 3:** Induction of peptide-specific CTLs from the PBMCs of HLA-A11, -A31, and -A33 cancer patients

	**Peptide**						
	**Lck_90–99_**	**Lck_449–458_**	**Lck_450–458_**	**Lck_452–461_**	**EBV**	**TRP-2**	**HIV**
**Patient**	**IFN-*γ* (pg ml^−1^)**						
*HLA-A11*							
1	10	—	1	—	**443**	8	—
2	**40**	15	—	**54**	3	**36**	—
3	**150**	**57**	**37**	—	—	2	—
4	**57**	9	**30**	—	2	**28**	—
5	—	8	—	—	1	2	—
6	**60**	**116**	**44**	**34**	**1000**	—	**41**
7	**87**	2	**232**	**133**	**583**	**140**	—
	5/7	2/7	4/7	3/7	3/7	3/7	1/7
							
*HLA*-*A31*							
8	15	6	—	26	11	4	—
9	22	17	**72**	15	**43**	17	—
10	**34**	**98**	**23**	30	**32**	**40**	—
11	**127**	**34**	**341**	11	**40**	**89**	—
12	**299**	**96**	**118**	**31**	12	—	—
	3/5	3/5	4/5	1/5	3/5	2/5	0/5
							
*HLA*-*A33*							
13	**151**	**55**	**28**	13	**48**	19	—
14	14	—	**24**	—	207	2	—
15	**61**	**60**	**105**	15	**39**	**157**	—
16	9	**100**	16	**26**	—	19	—
17	3	6	—	—	**67**	20	—
	2/5	3/5	3/5	1/5	3/5	1/5	0/5
Positive/total	10/17	8/17	11/17	5/17	9/17	6/17	1/17

CTL=cytotoxic T lymphocyte; HLA=human leukocyte antigen; IFN-*γ*=interferon-*γ*; PBMC=peripheral blood mononuclear cell.

The PBMCs from HLA-A11, -A31, and -A33 cancer patients were stimulated *in vitro* with the indicated Lck peptides.

On day 14, the cultured PBMCs were tested for their reactivity to C1R-A11, -A31, or -A33 cells, which were pre-pulsed with a corresponding peptide or the HIV peptide. The values represent the results of positive wells among four wells, and the background IFN-*γ* production in response to the HIV peptide was subtracted. Significant values (*P*<0.05 by two-tailed Student's *t*-test) are shown in bold.

## References

[bib1] Aizawa M (1986) The Proceedings of the 3rd Asia-Oceania Histocompatibility Conference pp 1090–1103. Oxford: Oxford University Press

[bib2] Amundadottir LT, Leder P (1998) Signal transduction pathways activated and required for mammary carcinogenesis in response to specific oncogenes. Oncogene 16: 737–746 9488037 10.1038/sj.onc.1201829

[bib3] Harada M, Matsueda S, Yao A, Ogata R, Noguchi M, Itoh K (2004) Prostate-related antigen-derived new peptides having the capacity of inducing prostate cancer-reactive CTLs in HLA-A2^+^ prostate cancer patients. Oncol Rep 12: 601–607 15289844

[bib4] Harashima N, Tanaka K, Sasatomi T, Shimizu K, Miyagi Y, Yamada A, Tamura M, Yamana H, Itoh K, Shichijo S (2001) Recognition of the Lck tyrosine kinase as a tumor antigen by cytotoxic T lymphocytes of cancer patients with distant metastases. Eur J Immunol 31: 323–332 11180095 10.1002/1521-4141(200102)31:2<323::aid-immu323>3.0.co;2-0

[bib5] Hida N, Maeda Y, Katagiri K, Takasu H, Harada M, Itoh K (2002) A simple culture protocol to detect peptide-specific cytotoxic T lymphocyte precursors in the circulation. Cancer Immunol Immunother 51: 219–228 12012109 10.1007/s00262-002-0273-7 PMC11032804

[bib6] Imai N, Harashima N, Ito M, Miyagi Y, Harada M, Yamada A, Itoh K (2001) Identification of Lck-derived peptides capable of inducing HLA-A2-restricted and tumor-specific CTLs in cancer patients with distant metastases. Int J Cancer 94: 237–242 11668504 10.1002/ijc.1461

[bib7] Imanishi T, Akazawa T, Kimura A (1992) Allele and haplotype frequencies for HLA and complement loci in various ethnic groups. In HLA 1991, Tsuji K, Aizawa M, Sasazuki T (eds), vol. 1, pp 1065–1220. Oxford: Oxford Scientific Publications

[bib8] Komatsu N, Shichijo S, Nakagawa M, Itoh K (2004) New multiplexed flow cytometric assay to measure anti-peptide antibody: a novel tool for monitoring immune responses to peptides used for immunization. Scand J Clin Lab Invest 64: 535–545 15370458 10.1080/00365510410007008

[bib9] Krystal GW, DeBerry CS, Linnekin D, Litz J (1998) Lck associates with and is activated by kit in a small cell lung cancer cell line: inhibition of SCF-mediated growth by the Src family kinase inhibitor PP1. Cancer Res 58: 4660–4666 9788619

[bib10] Lutz MP, Eber IBS, Flossmann-Kast BBM, Vogelmann R, Luhrs H, Friess H, Buchler W, Adler G (1998) Overexpression and activation of the tyrosine kinase Src in human pancreatic carcinoma. Biochem Biophys Res Commun 243: 503–508 9480838 10.1006/bbrc.1997.8043

[bib11] Marth JD, Cooper JA, King CS, Ziegler SF, Tinker DA, Overell RW, Krebs EG, Perlmutter RM (1988) Neoplastic transformation induced by an activated lymphocyte-specific protein tyrosine kinase (p56^lck^). Mol Cell Biol 8: 540–550 3352600 10.1128/mcb.8.2.540-550.1988 PMC363178

[bib12] Matsueda S, Takedatsu H, Yao A, Tanaka M, Noguchi M, Itoh K, Harada M (2005) Identification of peptide vaccine candidates for prostate cancer patients with HLA-A3 supertype alleles. Clin Cancer Res 11: 6933–6943 16203785 10.1158/1078-0432.CCR-05-0682

[bib13] McCracken S, Kim CS, Xu Y, Minden M, Miyamamto NG (1997) An alternative pathway for expression of p56^*lck*^ from type I promoter transcript in colon carcinoma. Oncogene 15: 2929–2937 9416836 10.1038/sj.onc.1201474

[bib14] Minami T, Matsueda S, Takedatsu H, Tanaka M, Noguchi M, Uemura H, Itoh K, Harada M (2007) Identification of SART3-derived peptides having the potential to induce cancer-reactive cytotoxic T lymphocytes from prostate cancer patients with HLA-A3 supertype alleles. Cancer Immunol Immunother 56: 689–698 16937115 10.1007/s00262-006-0216-9 PMC11030603

[bib15] Mine T, Sato Y, Noguchi M, Sasatomi T, Gouhara R, Tsuda N, Tanaka S, Shoumura H, Katagiri K, Rikimaru T, Shichijo S, Kamura T, Hashimoto T, Shirouzu K, Yamada A, Todo S, Itoh K, Yamana H (2004) Humoral responses to peptides correlated with overall survival in advanced cancer patients vaccinated with peptides based on pre-existing, peptide-specific cellular responses. Clin Cancer Res 10: 929–937 14871969 10.1158/1078-0432.ccr-1117-3

[bib16] Nakatsura T, Senju S, Ito M, Nishimura Y, Itoh K (2002) Cellular and humoral immune responses to a human pancreatic cancer antigen, coactosin-like protein, originally defined by the SEREX method. Eur J Immunol 32: 826–836 11870627 10.1002/1521-4141(200203)32:3<826::AID-IMMU826>3.0.CO;2-Y

[bib17] Ohkouchi S, Yamada A, Imai N, Mine T, harada K, Shichijo S, Maeda Y, Saijo Y, Nukiwa T, Itoh K (2002) Non-mutated tumor-rejection antigen peptides elicit type-I allergy in the majority of healthy individuals. Tissue Antigens 59: 259–272 12135424 10.1034/j.1399-0039.2002.590403.x

[bib18] Parker KC, Bednarek MA, Coligan JE (1994) Scheme for ranking potential HLA-A2 binding peptides based on independent binding of individual peptide side-chains. J Immunol 152: 163–175 8254189

[bib19] Rammensee HG, Friege T, Stevanovics S (1995) MHC ligands and peptides motifs. Immunogenetics 41: 178–228 7890324 10.1007/BF00172063

[bib20] Renkvist N, Castelli C, Robbins PF, Parmiani G (2001) A listing of human tumor antigens recognized by T cells. Cancer Immunol Immunother 50: 3–15 11315507 10.1007/s002620000169 PMC11036832

[bib21] Robinson D, He F, Pretlow T, Kung HJ (1996) A tyrosine kinase profile of prostate carcinoma. Proc Natl Acad Sci USA 93: 5958–5962 8650201 10.1073/pnas.93.12.5958 PMC39170

[bib22] Sato Y, Shomura H, Maeda Y, Mine T, Ueno Y, Akasaka Y, Kondo M, Takahashi S, Shinohara T, Katagiri K, Sato M, Okada S, Matsui K, Yamada A, Yamana H, Itoh K, Todo S (2003) Immunological evaluation of peptide vaccination for patients with gastric cancer based on pre-existing cellular response to peptide. Cancer Sci 94: 802–808 12967479 10.1111/j.1349-7006.2003.tb01522.x PMC11160207

[bib23] Sette A, Sidney J (1999) Nine major HLA class I supertypes account for the vast preponderance of HLA-A and -B polymorphism. Immunogenetics 50: 201–212 10602880 10.1007/s002510050594

[bib24] Sidney J, Grey HM, Southwood S, Celis E, Wentworth PA, del Guercio MF, Kubo RT, Chestnut RW, Sette A (1996) Definition of an HLA-A3-like supermotif demonstrates the overlapping peptide-binding repertoires of common HLA molecules. Hum Immunol 45: 79–93 8882405 10.1016/0198-8859(95)00173-5

[bib25] Veillette A, Bookman MA, Horak EM, Samelson LE, Bolen JB (1989) Signal transduction through the CD4 receptor involves the activation of the internal membrane tyrosine-protein kinase p56^*lck*^. Nature 338: 257–259 2784195 10.1038/338257a0

[bib26] Yamada A, Itoh K (2006) Personalized peptide vaccines: a new therapeutic modality for cancer. Cancer Sci 97: 970–976 16984371 10.1111/j.1349-7006.2006.00272.x PMC11158045

